# Non-linear relationship between lipid accumulation products and risk of diabetes in Japanese adults

**DOI:** 10.1038/s41598-024-78672-0

**Published:** 2024-11-07

**Authors:** Fubing Zha, Miaoling Chen, Linlin Shan, Jingpu Zhao, Changchun Cao, Yulong Wang

**Affiliations:** 1 Department of Rehabilitation, Shenzhen Second People’s Hospital, The First Affiliated Hospital of Shenzhen University, No.3002, Sungang West Road, Futian District, Shenzhen, 518000 Guangdong Province China; 2 Department of Rehabilitation, Shenzhen Second People’s Hospital Dapeng New District Nan’ao Hospital, No. 6, Renmin Road, Dapeng New District, Shenzhen, 518000 Guangdong Province China

**Keywords:** Lipid accumulation product, Triglyceride, Waist circumference, Diabetes mellitus, Non-linear, Endocrine system and metabolic diseases, Metabolic disorders, Endocrinology

## Abstract

The lipid accumulation product (LAP) is a novel, sex-specific indicator of lipid accumulation. While previous studies have investigated the relationship between LAP and diabetes, they have not specifically addressed potential sex differences in this association. The present study explores the potential sex-specific differences in the relationship between LAP and diabetes mellitus (DM) risk in the Japanese population. From 2004 to 2015, a total of 15,263 Japanese individuals without DM underwent physical assessments at Murakami Memorial Hospital. The association between LAP and incident diabetes was analyzed using Cox proportional hazards regression models, incorporating cubic spline functions and smooth curve fitting to identify potential non-linear relationships. Subgroup and sensitivity analysis assessments were conducted to validate the robustness of our findings. After adjusting for covariates, a positive association between LAP and DM risk was observed in both females (HR: 1.02, 95% CI: 1.00-1.03, *P* = 0.0138) and males (HR: 1.01, 95% CI: 1.00-1.01, *P* = 0.0314). A non-linear association between LAP and incident DM was identified for both sexes. The inflection points for LAP were 16.58 for females and 11.11 for males. In females, the HR to the left and right of the inflection point were 1.09 (95% CI: 1.02–1.17) and 1.01 (95% CI: 1.00-1.03), respectively. In males, the HR to the left and right of the inflection point were 0.92 (95% CI: 0.86–0.98) and 1.01 (95% CI: 1.00-1.01), respectively. Sensitivity analysis indicated a stronger association between LAP and DM risk. Elevated baseline LAP levels were associated with an increased risk of incident DM, with the relationship exhibiting non-linear characteristics. These findings suggest that maintaining lower LAP levels may help mitigate the risk of developing diabetes.

**Supplementary Information:**

The online version contains supplementary material available at 10.1038/s41598-024-78672-0.

## Introduction

Diabetes Mellitus (DM) is a persistent metabolic condition marked by high blood glucose levels. As reported by the International Diabetes Federation, about 463 million people aged 20–79 worldwide were diagnosed with diabetes in 2019, indicating a prevalence of 9.3%^1^. Additionally, the occurrence and prevalence of diabetes are rising among young and middle-aged populations^2^. This disorder is among the common metabolic conditions, leading to significant economic burdens for both individuals and countries^3^. Although DM is typically seen as irreversible, preventive strategies can significantly reduce its impact^4^. A thorough understanding of its risk factors is crucial for effective prevention and identification of DM.

Emerging research indicates that visceral fat plays a crucial role in the onset of DM and insulin resistance (IR)^5,6^. Common anthropometric measures of obesity, such as waist-to-hip ratio, waist circumference (WC), and body mass index (BMI), do not differentiate between subcutaneous and visceral fat^7^. A more promising predictor of metabolic syndrome has been identified-the lipid accumulation product (LAP), calculated from fasting triglyceride (TG) levels and WC measurements, which assesses visceral fat accumulation^8^. Several recent studies have found that increased LAP increases the risk of DM^9–11^. However, some studies have reached inconsistent conclusions. These studies found that elevated LAP levels have not consistently shown a strong link to a higher risk of type 2 diabetes (T2DM), suggesting that LAP may not be a reliable predictor of T2DM^12,13^. While previous studies have investigated the relationship between LAP and diabetes, they have not specifically addressed potential sex differences in this association. Considering the sex-specific differences in body fat percentage and distribution patterns, the relationship may vary between males and females^14^. Regrettably, there is a lack of comprehensive studies examining the sex-specific relationship between LAP and DM. Therefore, this study aims to clarify the association between LAP and DM in the Japanese population, taking sex differences into account.

## Methods

### Data source

Researchers have the ability to freely access and reference original datasets from the Dryad Digital Repository. In this research, we employed the raw data supplied by Takuro Okamura et al.^15^. This dataset was obtained from the Dryad repository. In compliance with Dryad’s terms of service, the data were used for secondary analysis. Our study conducted a secondary examination utilizing publicly accessible data from medical examination programs.

### Study participants

In the initial study, written informed consent was provided by all participants, and approval was obtained from the Clinical Research Ethics Committee at Murakami Memorial Hospital^15^. Given that the current study involves a secondary analysis of existing data, there was no need to obtain informed consent or additional ethical approval. The original research adhered to the principles of the Declaration of Helsinki, ensuring that all procedures described in the Declarations section were conducted in accordance with relevant regulations and guidelines.

**Fig. 1:**
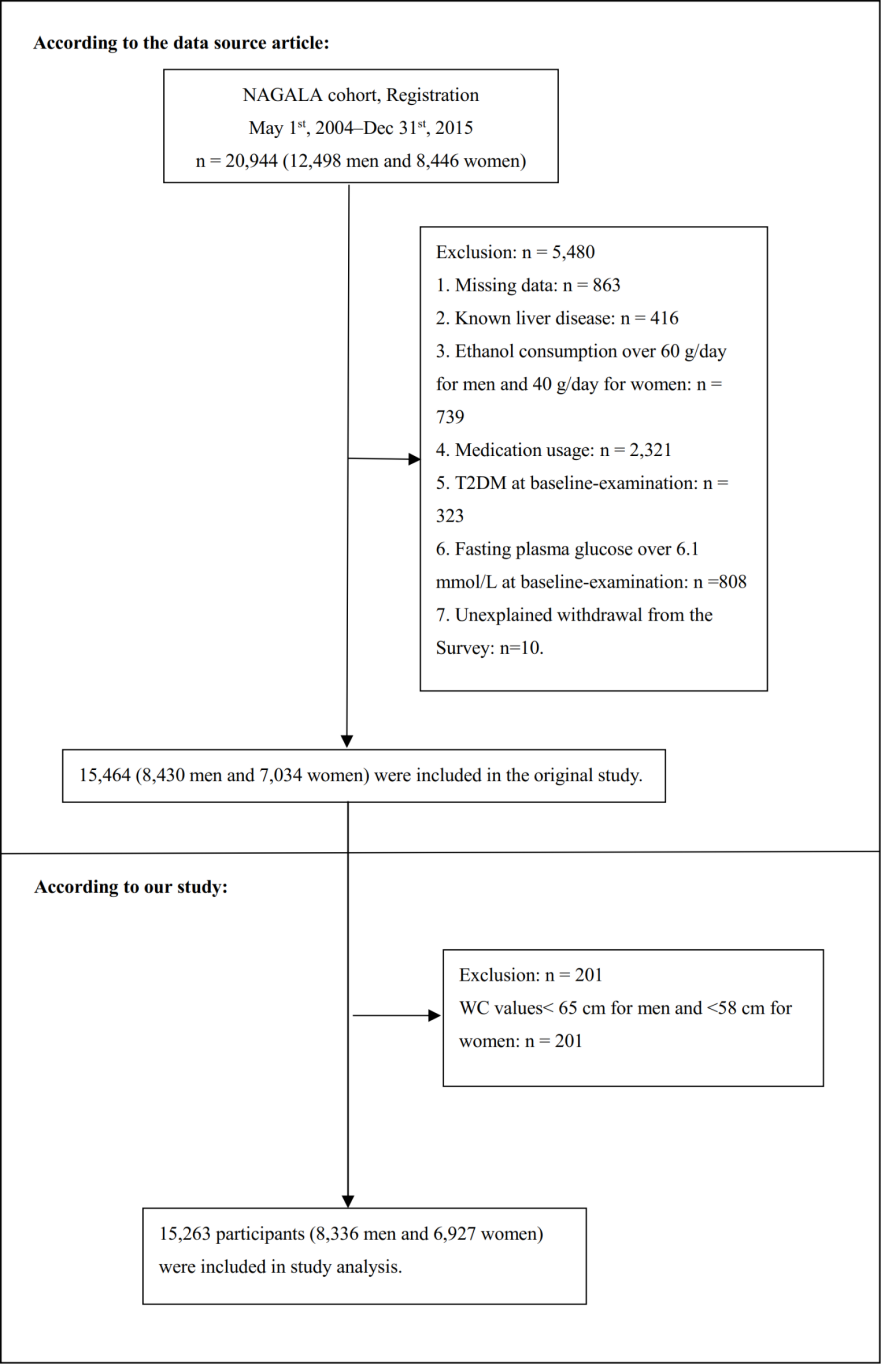
Study population.

This research utilized open-source data from the NAGALA database, derived from a secondary analysis of a medical examination program. The center where these programs were conducted, established in 1994, performed over 8000 medical examinations annually, with 60% of participants undergoing one to two exams per year. Due to the high frequency of repeated examinations, the original study cohort included all participants who underwent at least two evaluations between 2004 and 2015. In the original study, 5,480 participants out of 20,944 Japanese participants were excluded based on the following criteria: (1) missing data for variables; (2) pre-existing liver diseases, such as hepatitis C or hepatitis B; (3) ethanol consumption over 60 g/day for men and 40 g/day for women; (4) medication usage; (5) diabetes; (6) fasting plasma glucose (FPG) levels ≥ 6.1 mmol/L; (7) unexplained withdrawal from the survey. Consequently, 15,464 participants were included in the original study. Our study further excluded 201 participants for the following reasons: WC values < 58 cm for females, and < 65 cm for males. Finally, our study included 15,263 eligible participants. Figure 1 detailed the selection process for all the participants.

### Covariates

We choose covariates using clinical expertise and previous research results^9–11^. The covariates included: (1) continuous variables: alanine aminotransferase (ALT), total cholesterol (TC), systolic blood pressure (SBP), glycosylated hemoglobin (HbA1c), gamma-glutamyl transferase (GGT), diastolic blood pressure (DBP), high-density lipoprotein cholesterol (HDL-C), age, aspartate aminotransferase (AST), FPG, and body mass index (BMI); (2) categorical variables: smoking status, exercise habits, alcoholic intake, and sex. The original study gathered data on each individual’s medical history and lifestyle through a standardized self-management questionnaire. Trained professionals accurately measured WC, weight, height, and blood pressure. Laboratory test results were obtained using consistent procedures by the original study team under controlled conditions.

### LAP

The calculation uses the following formulas: TG (mmol/L) × (WC (cm) − 65) for men and TG (mmol/L) × (WC (cm) − 58) for women, where WC values was ≥ 65 cm for males and ≥ 58 cm for females^16^.

### Diagnosis of incident diabetes

Diabetes was defined as having self-reported history, glycosylated hemoglobin levels more than 6.5%, or FPG ≥ 7.0 mmol/L^17^. For participants with a diagnosis of diabetes based on FPG or HbA1c levels, the incidence time was documented as the date of the visit when the diagnostic criteria were first satisfied. For participants with a self-reported diabetes, the incidence time was recorded as the date of the visit when they initially reported the diagnosis. The follow-up period was calculated from the baseline visit to the date of diabetes diagnosis or the date of the last visit, whichever occurred first.

### Statistical analysis

We performed statistical analyses using Empower-Stats and SPSS 22.0. Baseline characteristics of participants were examined across LAP quartiles. Continuous variables with skewed distributions were presented as medians (quartiles), while those with normal distributions were shown as means ± standard deviations. Quantitative variables were described through counts and percentages (%). To explore variations in baseline characteristics across groups, categorical variables were analyzed using the chi-square test, while continuous variables were compared using Student’s t-test (normal distribution) and the Mann-Whitney U test (non-normal distribution).

Univariate Cox regression was employed to evaluate the impact of individual variables on diabetes risk. To further elucidate the relationship between LAP and diabetes risk, we conducted a multivariate Cox regression analysis. According to the findings of previous studies and the application of a directed acyclic graph (Supplementary Fig. S1), SBP, DBP, and HbA1c were excluded due to collinearity. Consequently, we identified the minimally sufficient adjustment set for our analysis. Based on the results of the collinearity screening within the minimally sufficient adjustment set, no variables were excluded from the final multivariate Cox proportional hazards regression equation (Supplementary Table S1).

Four models were developed: Model 1 (unadjusted), Model 2 (adjusted for age, sex alcohol intake, smoking status, and exercise habits), Model 3 (adjusted for age, sex alcohol intake, smoking status, exercise habits and BMI) and Model 4 (adjusted for age, sex alcohol intake, smoking status, exercise habits, BMI, ALT, AST, GGT, HDL-C, TC, and FPG). Throughout the study, we documented HR and 95% confidence intervals (CI).

Given that LAP is a continuous variable, we explored potential nonlinear relationships between LAP and diabetes using Cox proportional hazards regression with cubic spline functions and smooth curve fitting. For nonlinear associations, a two-piecewise Cox proportional hazards regression model identified the inflection point^18^. The optimal model for LAP’s association with diabetes was determined using log-likelihood ratio tests.

We conducted rigorous sensitivity analyses to ensure the robustness of our findings. LAP was categorized by quartiles, and a P-value for trend was calculated to validate results and investigate nonlinearity. Hypertension and advanced age are well-documented risk factors for diabetes, as established by numerous scholarly studies^19,20^. To assess the robustness of the relationship between LAP and diabetes risk, additional sensitivity analyses were performed, excluding individuals with elevated blood pressure (DBP ≥ 90 mmHg or SBP ≥ 140 mmHg)^21^ or those aged ≥ 45 years^22^.

Subgroup analysis using the Cox proportional hazard model was also performed, with variables including DBP, SBP, alcohol intake, exercise habits, smoking status, BMI, and sex. Stratification was based on medians or clinical cut points^23^, converting variables such as SBP (< 140, ≥ 140 mmHg), and DBP (< 90, ≥ 90 mmHg)^21^ into categorical factors. Each stratum underwent a fully adjusted analysis, except for the stratification factor. Likelihood ratio tests were conducted to assess subgroup interactions^24,25^. The study adhered to the STROBE statement for all outcomes^26,27^. The findings were considered statistically significant if the two-sided P value was below 0.05.

## Results

### Participant characteristics and the incidence rate of DM

Table 1 provides a detailed overview of clinical, biochemical, and additional parameters measured across all subjects. The final analysis encompassed 15,263 individuals, and the median follow-up duration of our study was 5.39 years. The mean age of subjects in the study was 43.73 ± 8.88 years, with males comprising 54.62% of the cohort. Subjects were divided into quartiles based on LAP values as follows: Q1 (LAP ≤ 4.89), Q2 (4.89 < LAP ≤ 9.95), Q3 (9.95 < LAP ≤ 9.67) and Q4 (LAP > 9.67). Those in the highest LAP quartile had elevated levels of DBP, BMI, GGT, TG, SBP, ALT, age, HbA1c, AST, alcoholic intake, TC, WC, and FPG, along with higher proportions of male participants and smokers. Conversely, they exhibited lower HDL-C levels and were less likely to engage in regular exercise. This phenomenon was consistently observed when LAP quartiles were grouped separately for males and females (Supplementary Table S2A and B).

**Table 1:** The characteristics of participants and incidence rate of diabetes.

Characteristic	Total	Q1 (LAP ≤ 4.89)	Q2 (4.89 < LAP ≤ 9.95)	Q3 (9.95 < LAP ≤ 19.67	Q4 (LAP > 19.67)	*P*-value
Participants	15,263	3813	3811	3820	3819	
Sex						< 0.001
Female	6927 (45.38%)	2524 (66.19%)	2027 (53.19%)	1493 (39.08%)	883 (23.12%)	
Male	8336 (54.62%)	1289 (33.81%)	1784 (46.81%)	2327 (60.92%)	2936 (76.88%)	
Age(years)	43.73 ± 8.88	40.65 ± 8.46	43.13 ± 8.80	45.23 ± 8.74	45.91 ± 8.57	< 0.001
Alcoholic intake (g/wk)	42 (37–50)	1 (0-23.38)	1 (0–60)	2.8 (0–84)	12 (1-108)	< 0.001
Smoking status						< 0.001
Never-smoker	8898 (58.30%)	2761 (72.41%)	2428 (63.71%)	2043 (53.48%)	1666 (43.62%)	
Ex-smoker	2932 (19.21%)	470 (12.33%)	660 (17.32%)	868 (22.72%)	934 (24.46%)	
Current-smoker	3433 (22.49%)	582 (15.26%)	723 (18.97%)	909 (23.80%)	1219 (31.92%)	
Exercise habits						< 0.001
No	12,592 (82.50%)	3095 (81.17%)	3102 (81.40%)	3152 (82.51%)	3243 (84.92%)	
Yes	2671 (17.50%)	718 (18.83%)	709 (18.60%)	668 (17.49%)	576 (15.08%)	
SBP (mmHg)	114.63 ± 14.94	107.14 ± 12.70	111.55 ± 13.23	116.71 ± 13.85	123.11 ± 14.95	< 0.001
DBP (mmHg)	71.67 ± 10.50	66.44 ± 8.84	69.44 ± 9.48	72.94 ± 9.79	77.84 ± 10.25	< 0.001
BMI (kg/m^2^**)**	22.18 ± 3.10	19.41 ± 1.69	21.22 ± 1.87	22.82 ± 2.18	25.27 ± 2.94	< 0.001
WC (cm)	76.70 ± 8.93	67.26 ± 4.61	73.88 ± 4.93	79.34 ± 5.43	86.29 ± 6.93	< 0.001
ALT (IU/L)	17 (13–23)	14 (11–18)	15 (12–19)	18 (13-23.25)	23 (17–33)	< 0.001
AST (IU/L)	17 (14–21)	16 (13–19)	16 (13–20)	17 (14–21)	20 (16–24)	< 0.001
GGT (IU/L)	15 (11–22)	12 (10–15)	13 (11–18)	16 (12–23)	23 (16–35)	< 0.001
HDL-C (mmol/L)	1.46 ± 0.40	1.69 ± 0.39	1.57 ± 0.37	1.40 ± 0.35	1.17 ± 0.29	< 0.001
TG (mmol/L)	0.73 (0.50–1.13)	0.42 (0.32–0.55)	0.59 (0.47–0.75)	0.85 (0.68–1.05)	1.48 (1.15–1.94)	< 0.001
TC (mmol/L)	5.13 ± 0.86	4.76 ± 0.78	4.98 ± 0.81	5.22 ± 0.81	5.54 ± 0.86	< 0.001
HbA1c (%)	5.17 ± 0.32	5.11 ± 0.30	5.14 ± 0.31	5.19 ± 0.32	5.25 ± 0.34	< 0.001
FPG (mmol/L)	5.16 ± 0.41	4.97 ± 0.39	5.09 ± 0.40	5.22 ± 0.38	5.37 ± 0.37	< 0.001
DM						< 0.001
No	14,890 (97.56%)	3786 (99.29%)	3774 (99.03%)	3747 (98.09%)	3583 (93.82%)	
Yes	373 (2.44%)	27 (0.71%)	37 (0.97%)	73 (1.91%)	236 (6.18%)	
Cumulative incidence (%) (95% CI)	2.44 (2.20–2.69)	0.71 (0.44–0.97)	0.97 (0.66–1.28)	1.91 (1.48-2,35)	6.18 (5.42–6.94)	< 0.001
Per 100,000 person-year	404.23	116.08	163.41	318.36	1,006.74	< 0.001

Values are presented as n (%) or mean ± SD or median (quartile).

LAP: lipid accumulation product; BMI: body mass index; WC: waist circumference; SBP: systolic blood pressure; DBP: diastolic blood pressure; ALT: alanine aminotransferase; AST: aspartate aminotransferase; GGT: gamma-glutamyl transferase; HDL-C: high-density lipoprotein cholesterol; TC: total cholesterol; TG: triglycerides; HbA1c: hemoglobin A1c; FPG: fasting plasma glucose; CI: confidence interval; DM: diabetes mellitus.

Table 1 also outlines that 373 individuals developed DM during the follow-up period, corresponding to an overall incidence rate of 2.44% (2.20-2.69%). The prevalence rates were 0.71% (0.44-0.97%), 0.97% (0.66-1.28%), 1.91% (1.48%-2,35%) and 6.18% (5.42-6.94%) for the first, second, third, and fourth LAP groups, respectively. The cumulative incidence rates per 100,000 person-years were 404.23 for the total population, and 116.08, 163.41, 318.36, and 1,006.74 for the first, second, third, and fourth LAP groups, respectively. Higher LAP levels were associated with increased diabetes incidence and cumulative prevalence. This phenomenon was consistently observed in both males and females (Supplementary Table S2A and B).

### The results of univariate analysis of factors associated with diabetes

**Fig. 2:**
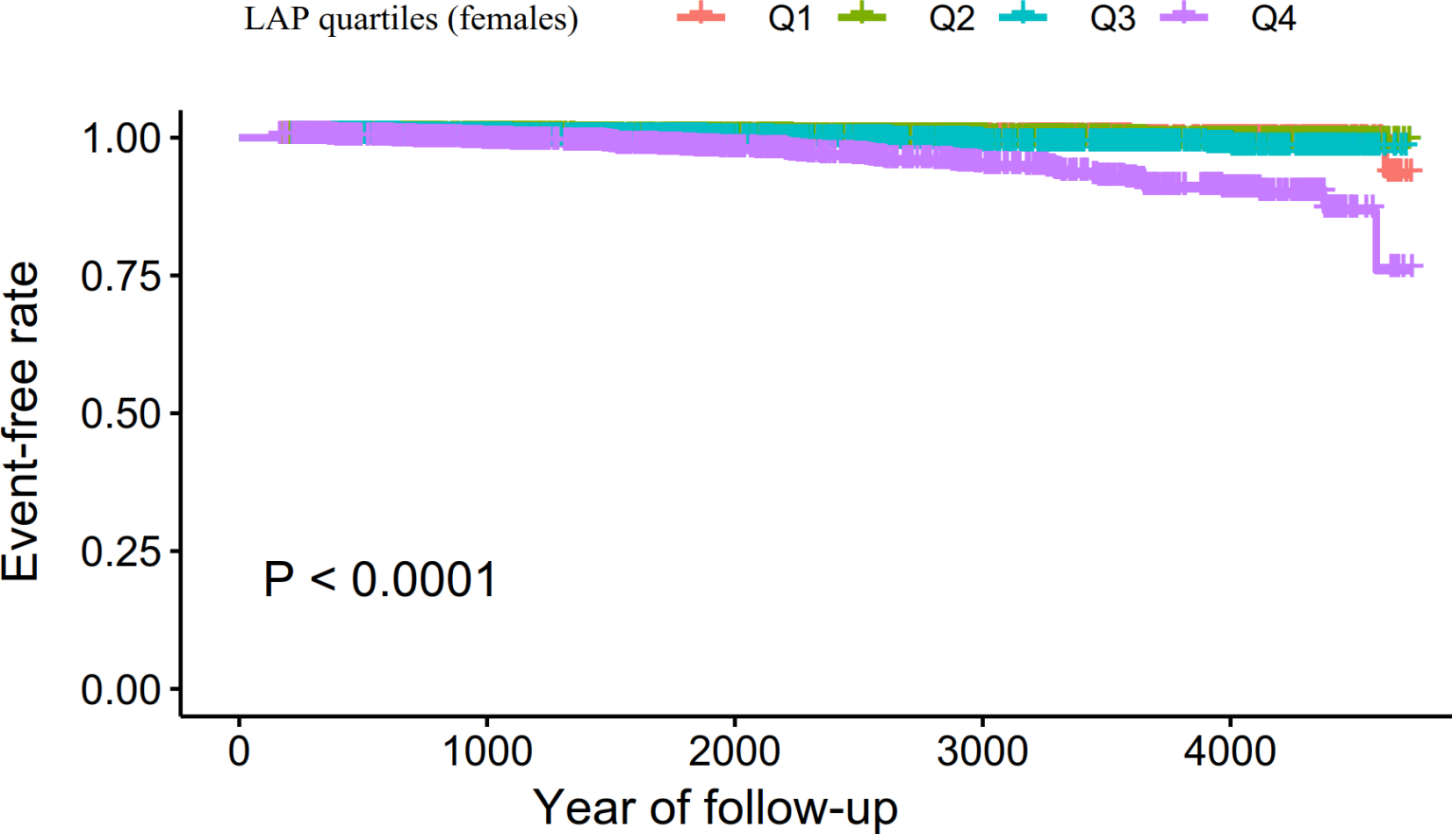
Kaplan–Meier event-free survival curve in females. Kaplan–Meier analysis of incident diabetes in females based on LAP quartiles (log-rank, *P* < 0.0001).

**Fig. 3:**
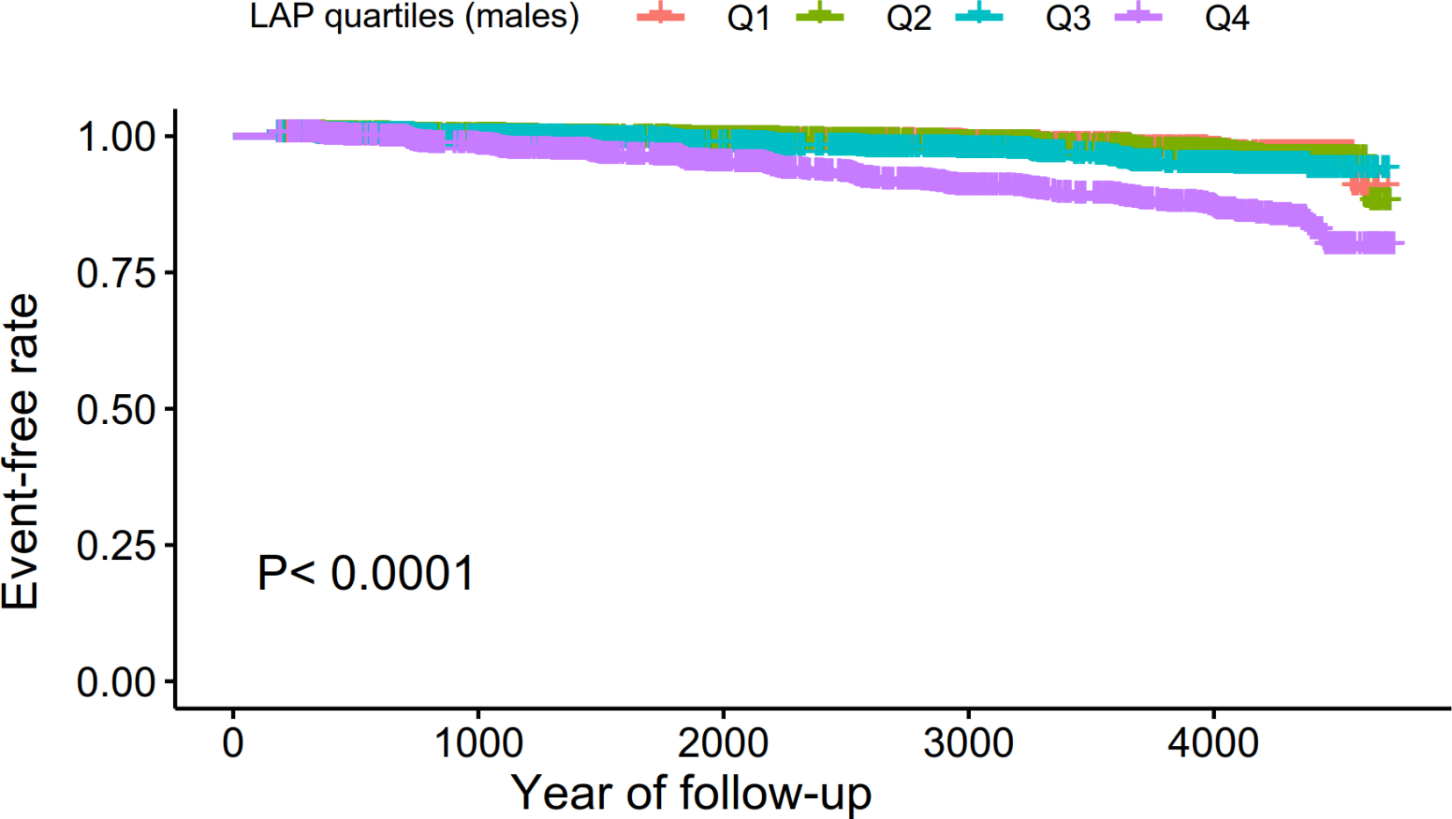
Kaplan–Meier event-free survival curve in males. Kaplan–Meier analysis of incident diabetes in males based on LAP quartiles (log-rank, *P* < 0.0001).

Supplementary Table S3 summarizes the univariate analysis results, indicating positive associations between DBP, BMI, GGT, TG, SBP, ALT, age, HbA1c, AST, alcoholic intake, TC, WC, FPG, and smoking with diabetes risk. HDL-C was inversely related to diabetes risk. Males exhibited a higher propensity for diabetes compared to females. Kaplan-Meier curves in Figs. 2 and 3 illustrate a significant difference in diabetes-free survival among the four LAP groups in females and males (*P* < 0.0001), with higher LAP levels correlating with decreased diabetes-free survival.

### The results of the association between LAP and DM risk

As LAP met the proportional hazards assumption, the association between the LAP and diabetes risk was evaluated using the Cox proportional hazards regression model (Supplementary Table S4 and Supplementary Fig. S2). Table 2 presents the results of Cox proportional hazard regression analyses, highlighting the relationship between LAP and diabetes incidence. In Model 1 (unadjusted), LAP showed a positive association with diabetes (HR: 1.04, 95% CI: 1.04–1.05, *P* < 0.0001 for females; HR: 1.02, 95% CI: 1.02–1.03, *P* < 0.0001 for males). This trend persisted in Model 2 (adjusted for age, sex, alcohol intake, smoking status, and exercise habits) with HR of 1.04 (95% CI: 1.03–1.05, *P* < 0.0001) for females and 1.02 (95% CI: 1.02–1.03, *P* < 0.0001) for males. In Model 3 (adjusted for age, sex, alcohol intake, smoking status, exercise habits and BMI), LAP also showed a positive association with diabetes (HR: 1.03, 95% CI: 1.01–1.04, *P* < 0.0001 for females; HR: 1.01, 95% CI: 1.01–1.02, *P* < 0.0001 for males). In Model 4 (adjusted for age, sex, alcohol intake, smoking status, exercise habits, BMI, ALT, AST, GGT, HDL-C, TC, and FPG), each unit increase in LAP raised the risk of DM by 2% and 1% for females and males (HR: 1.02, 95% CI: 1.00-1.03, *P* = 0.0138 for females; HR: 1.01, 95%CI: 1.00-1.01, *P* = 0.0314 for males).

**Table 2:** Relationship between LAP and incident diabetes in different models.

Variable	Model 1 (HR,95% CI, *P*)	Model 2 (HR,95% CI, *P*)	Model 3 (HR,95% CI, *P*)	Model 4 (HR,95% CI, *P*)
Total LAP	1.03 (1.03, 1.03) < 0.0001	1.03 (1.02, 1.03) < 0.0001	1.01 (1.01, 1.02) < 0.0001	1.01 (1.00, 1.01) 0.0084
Total LAP (quartile)				
Q1	Ref.	Ref.	Ref.	Ref.
Q2	1.43 (0.87, 2.34) 0.1595	1.23 (0.75, 2.02) 0.4199	0.85 (0.52, 1.41) 0.5388	0.64 (0.38, 1.07) 0.0874
Q3	2.77 (1.78, 4.31) < 0.0001	2.12 (1.35, 3.32) 0.0011	1.10 (0.69, 1.75) 0.6959	0.63 (0.39, 1.03) 0.0650
Q4	8.60 (5.78, 12.81) < 0.0001	5.94 (3.92, 9.01) < 0.0001	1.94 (1.21, 3.12) 0.0058	0.74 (0.44, 1.24) 0.2512
P for trend	< 0.0001	< 0.0001	< 0.0001	0.7429
Female LAP	1.04 (1.04, 1.05) < 0.0001	1.04 (1.03, 1.05) < 0.0001	1.03 (1.01, 1.04) < 0.0001	1.02 (1.00, 1.03) 0.0138
Female LAP (quartile)				
Q1	Ref.	Ref.	Ref.	Ref.
Q2	0.92 (0.28, 3.00) 0.8843	0.84 (0.26, 2.76) 0.7759	0.66 (0.20, 2.18) 0.4952	0.67 (0.20, 2.22) 0.5128
Q3	2.41 (0.90, 6.43) 0.0789	2.03 (0.76, 5.43) 0.1591	1.22 (0.45, 3.33) 0.7002	1.00 (0.36, 2.77) 0.9973
Q4	13.59 (5.88, 31.42) < 0.0001	10.22 (4.35, 23.99) < 0.0001	3.72 (1.43, 9.65) 0.0070	1.95 (0.70, 5.42) 0.1988
P for trend	< 0.0001	< 0.0001	0.0001	0.0543
Male LAP	1.02 (1.02, 1.03) < 0.0001	1.02 (1.02, 1.03) < 0.0001	1.01 (1.01, 1.02) < 0.0001	1.01 (1.00, 1.01) 0.0314
Male LAP (quartile)				
Q1	Ref.	Ref.	Ref.	Ref.
Q2	1.06 (0.65, 1.71) 0.8208	0.98 (0.61, 1.60) 0.9473	0.71 (0.43, 1.15) 0.1664	0.54 (0.33, 0.88) 0.0138
Q3	1.63 (1.05, 2.53) 0.0287	1.49 (0.96, 2.31) 0.0769	0.83 (0.52, 1.31) 0.4186	0.50 (0.31, 0.82) 0.0057
Q4	4.73 (3.22, 6.95) < 0.0001	4.20 (2.86, 6.18) < 0.0001	1.63 (1.03, 2.56) 0.0351	0.70 (0.42, 1.18) 0.1783
P for trend	< 0.0001	< 0.0001	0.0004	0.8172

Model 1: we did not adjust for any covariants.

Model 2: we adjusted for sex, age, alcoholic intake, smoking status, and exercise habits.

Model 3: we adjusted for sex, age, alcoholic intake, smoking status, exercise habits, and BMI.

Model 4: we adjusted for sex, age, alcoholic intake, smoking status, exercise habits, BMI, ALT, AST, GGT, HDL-C, TC, and FPG.

Note: The models were not adjusted for sex variables in both male and female models.

HR: hazard ratio; CI: confidence interval; Ref.: Reference; LAP: lipid accumulation product.

Moreover, the analysis revealed a progressive increase in the HR across the quartiles when using the first quartile (Q1) of LAP as the reference point. The adjusted HR (95% CI) was 1.95 (0.70, 5.42) among females and 0.70 (0.42, 1.18) among males in the Q4 group compared with the Q1 group.

### Sensitive analysis

To validate our results, we used extensive sensitivity analyses. Excluding participants with elevated blood pressure (DBP ≥ 90 mmHg or SBP ≥ 140 mmHg) maintained a positive association between LAP and DM (HR = 1.01, 95% CI: 1.01–1.02, *P* = 0.0006) (Supplementary Table S5, Model 5). Similarly, excluding participants aged ≥ 45 years showed consistent results, with LAP remaining positively associated with diabetes risk after adjusting for multiple covariates (HR = 1.01, 95% CI: 1.00-1.02, *P* = 0.0111) (Supplementary Table S5, Model 6). These analyses underscore the robustness of our findings.

### The analyses of the non-linear association

**Fig. 4:**
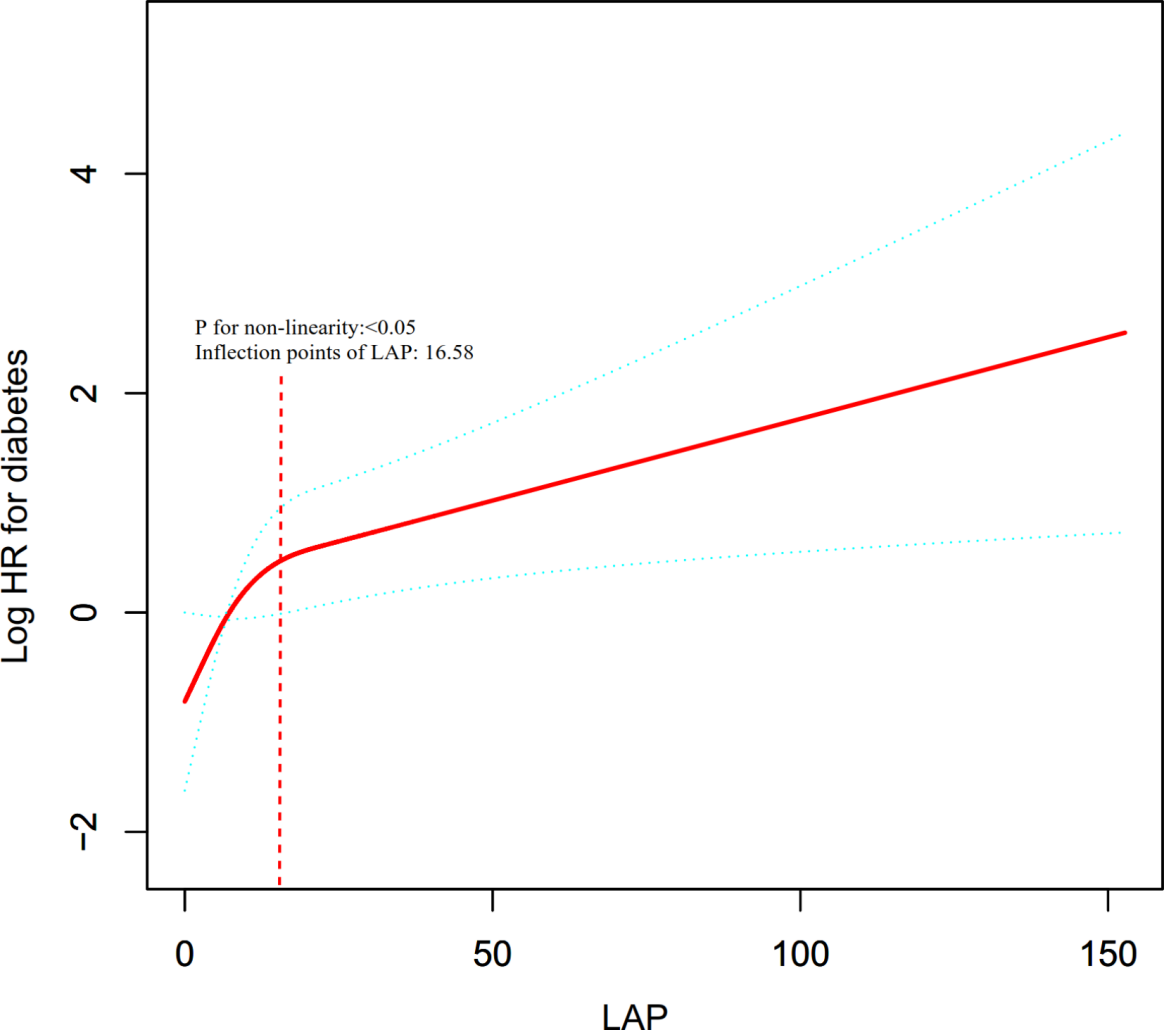
The nonlinear relationship between LAP ratio and incident diabetes in females. The nonlinear relationship was detected after adjusting for age, alcoholic intake, smoking status, exercise habits, BMI, ALT, AST, GGT, HDL-C, TC, and FPG.

**Fig. 5:**
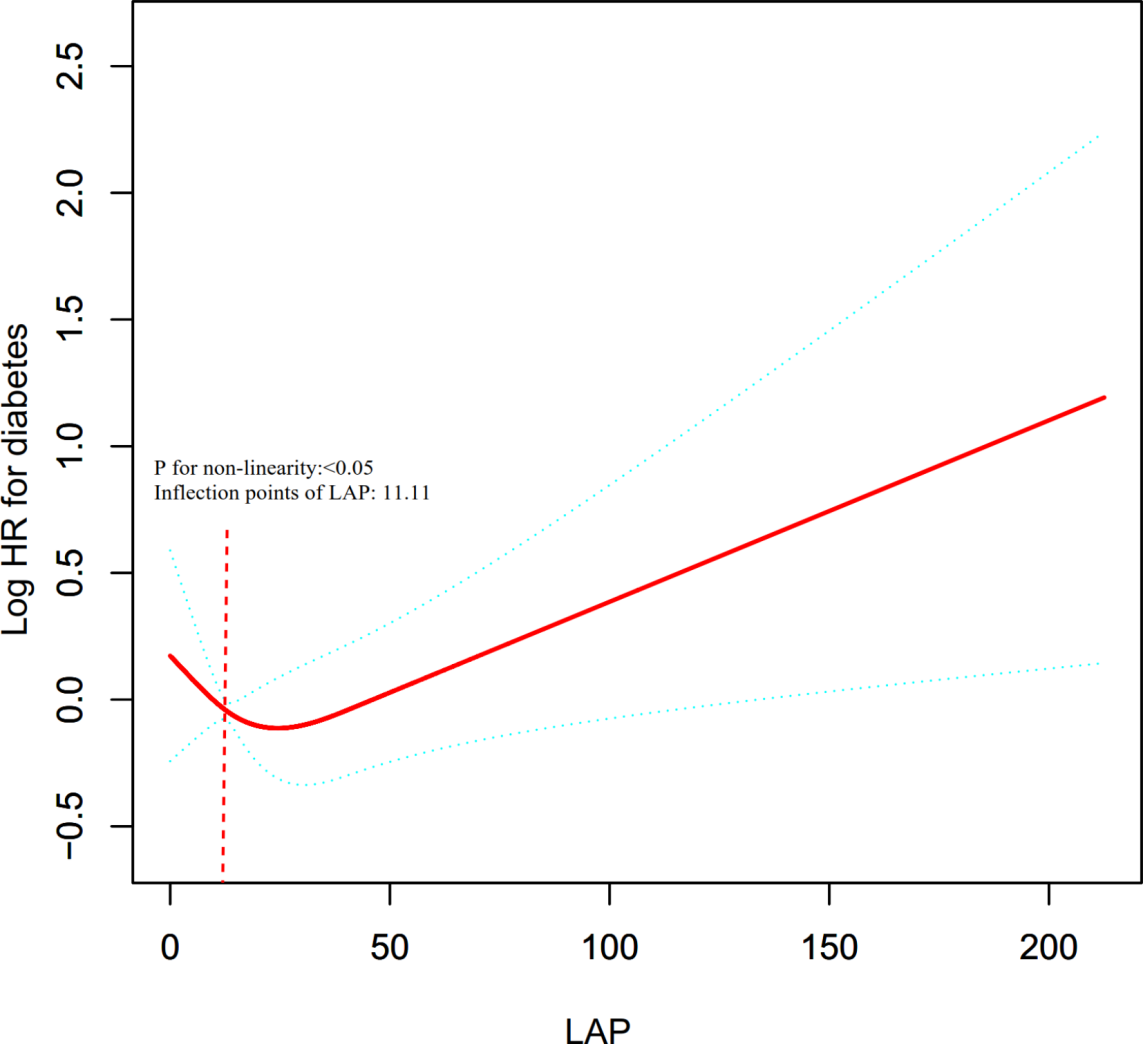
The nonlinear relationship between LAP ratio and incident diabetes in males. The nonlinear relationship was detected after adjusting for age, alcoholic intake, smoking status, exercise habits, BMI, ALT, AST, GGT, HDL-C, TC, and FPG.

Table 3; Figs. 4 and 5 indicate a nonlinear association between LAP and DM in both sexes, even after adjusting for confounders. The two-piecewise Cox regression model identified inflection points at 16.58 for females and 11.11 for males, with the log-likelihood ratio test yielding a significance level (*P* = 0.025 for females, *P* = 0.011 for males). For females, the HR was 1.01 (95% CI: 1.00-1.03) above the inflection point and 1.09 (95% CI: 1.02–1.17) below it. For males, For females, the HR was 1.01 (95% CI: 1.00-1.01) above the inflection point and 0.92 (95% CI: 0.86–0.98) below it.

**Table 3:** The result of the two-piecewise Cox proportional hazards regression model by sex.

Incident DM (female)	HR (95% CI),	*P*
Fitting model by standard linear regression	1.02 (1.00, 1.03) 0.0138	0.0206
Fitting model by two-piecewise Cox proportional hazards regression		
The inflection point of LAP	16.58	
≤16.58	1.09 (1.02, 1.17)	0.0091
>16.58	1.01 (1.00, 1.03)	0.0311
P for the log-likelihood ratio test	0.025	
Incident DM (male)		
Fitting model by standard linear regression	1.01 (1.00, 1.01)	0.0314
Fitting model by two-piecewise Cox proportional hazards regression		
The inflection point of LAP	11.11	
≤ 11.11	0.92 (0.86, 0.98)	0.0138
>11.11	1.01 (1.00, 1.01)	0.0271
P for the log-likelihood ratio test	0.011	

We adjusted for age, alcoholic intake, smoking status, exercise habits, BMI, ALT, AST, GGT, HDL-C, TC, and FPG.

HR: hazard ratios; CI: confidence; DM: diabetes mellitus; LAP: lipid accumulation product.

### The results of the subgroup analysis

Subgroup analysis explored potential modifiers of the LAP-diabetes relationship, considering factors such as DBP, sex, alcohol intake, SBP, exercise habits, and smoking status (Supplementary Table S6). DBP, and alcohol intake appeared to modify the relationship (interaction P value < 0.05), with a stronger association in individuals with no alcohol intake and DBP < 140 mmHg.

## Discussion

In this retrospective cohort analysis of 15,263 Japanese subjects, we demonstrated a link between increased LAP levels and the onset of diabetes. The investigation revealed a non-linear association between LAP levels and new-onset DM. Additionally, DBP, and alcohol intake emerged as a potential modifier of the association between LAP and DM risk, with the association being significantly more pronounced in individuals with no alcohol intake and DBP < 140 mmHg.

The calculation of LAP, which theoretically reflects the buildup of visceral fat, is based on WC and fasting TG values^8^. In comparison to conventional obesity indices (WC, BMI, and waist-to-height ratio), several studies found that LAP demonstrated a stronger predictive ability in diabetes, impaired fasting glucose, and metabolic syndrome^28–30^. In cross-sectional research of 215,651 Chinese adults, it was discovered that women had better LAP prediction accuracy than men^30^. In a retrospective analysis of 15,717 rural Chinese individuals, after adjusting for family income, family history of T2DM, age, alcoholic intake, physical activity, education, smoking status, and sex, Minrui Xu et^31^. found that compared with the Q1 group, the type 2 diabetes risk of LAP in the Q4 group was 8.063 times and 6.298 times higher in women and men (HR: 8.063, 95% CI: 5.645–11.516 for women, and HR: 6.298, 95% CI: 4.911–8.077 for men), respectively. Wang et al.^13^ conducted a study involving 11,113 Chinese individuals from a rural area over six years. They found that participants in the fourth quartile of LAP had a 5.02-fold increased risk for males and a 6.49-fold increased risk for females of developing diabetes, compared to those in the first quartile. In a retrospective study involving 7708 Koreans aged 40–69, after controlling for confounding covariates, the fourth quartile had a 2.47-fold for men and a 2.44-fold risk for women of getting diabetes compared with one quartile of LAP (HR: 2.47, 95% CI: 1.82–3.34 for men, and HR: 2.44, 95% CI: 1.82–3.26 for women)^32^. Besides, in a cross-sectional study involving 10,170 Japanese workers compared subjects without a high LAP, the odds ratio for DM in those with a high LAP was 7.40 (95% CI: 5.10-10.75) in males and 19.09 (95% CI:6.57–55.50) in females after adjusting for regular exercise, alcoholic intake, smoking, and age^33^. However, Nusrianto et al.^12^. found that high LAP was not associated to T2DM risk after adjustment for statin consumption, angiotensin-converting enzyme inhibitor consumption, other anti-hypertensive drugs, smoking status, age, family history of T2DM, and hypertension. In addition, Wang et al.^13^ also found that LAP might not be a cheap indicator for predicting T2DM. After adjusted for confounding factors, our retrospective cohort study showed that each unit increase in LAP increased the risk of diabetes by 2% in females and 1% in males. Furthermore, the results of the sensitivity analyses demonstrated that this association was still observed in Japanese subjects with normal blood pressure and age < 45 years. Based on the findings, a clinical LAP-level intervention guideline was developed with the purpose of reducing the risk of DM.

Moreover, our analysis revealed a nonlinear association between LAP and DM risk in both sexes after adjusting for confounding factors. The present research identified the LAP inflection point using a two-piecewise Cox proportional hazards regression model. In the group of females, a one-unit rise in LAP level when the LAP level was below 16.58 was associated with a 9% increase in HR for DM. A one-unit increase in LAP was linked to a 1% increase in the risk of DM when the LAP was greater than 16.58. In the group of males, when the LAP level was greater than 11.11, a one-unit increase in LAP level was associated with a 1% increase in HR for DM. A one-unit increase in LAP was linked to a 8% decrease in the risk of DM when the LAP was below 11.11. Our results suggest that sex-specific interventions may be necessary to optimize diabetes prevention strategies. For women, early intervention strategies aimed at controlling LAP before it reaches the inflection point may be particularly effective. Conversely, for men, interventions focused on lowering LAP near the inflection point may be crucial in minimizing diabetes risk. Additionally, public health policies could be adapted to incorporate sex-specific LAP thresholds into diabetes screening and prevention programs.

The exact process by which LAP causes diabetes to appear and progress is still unknown. High levels of TG and abdominal obesity may be linked to the etiology of DM^34–37^. Visceral adipocytes stimulate the production of several cytokines, including interleukin-6 and tumor necrosis factor-alpha, in patients with abdominal obesity, which encourages macrophage infiltration and persistent inflammation^38,39^. Adipocytes release adipose factor chemokine simultaneously, which affects lipid and carbohydrate metabolism^40,41^. Chronic inflammation can interfere with the signal transduction pathways of nearby cells, including B-regulatory cells, macrophages, eosinophils, and T cells, which can result in insulin resistance and eventually encourage the development of DM^41^. Much evidence has shown that higher levels of TG in muscle and liver may increase free fatty acids and their flux from adipose to non-adipose tissue, disrupting glucose metabolism^42,43^. Higher levels of TG increase fatty acid oxidation, which restricts insulin-stimulated glucose utilization, leading to a decrease in the absorption of glucose by the muscles and a reduction in the production of glycogen in the liver^44^.

Our study boasts several significant strengths. Firstly, we found the non-linear relationship, enabling us to identify the optimal inflection point for LAP’s influence on diabetes. Secondly, our results underwent thorough statistical adjustments to minimize confounding effects, thereby enhancing their reliability. Thirdly, the robustness of our conclusions was validated through extensive sensitivity analyses, which included LAP transformation and reassessment of the LAP-diabetes relationship after excluding participants with high blood pressure or those aged 45 years and above. Fourthly, our subgroup analysis revealed potential risk factors affecting the LAP-diabetes association.

Nevertheless, our study has certain limitations. Firstly, the research was confined to a Japanese cohort, which may limit the generalizability of the findings to other ethnic and geographical populations. Secondly, the study excluded participants with heavy alcohol consumption, viral hepatitis, drug use or missing data for variables, which may have introduced bias into our results. In future research, we plan to conduct original studies that include a broader population, encompassing individuals with heavy alcohol consumption, viral hepatitis, drug use or missing data for variables, to further explore the relationship between LAP and DM. Thirdly, as with any retrospective study, unmeasured or uncontrolled confounding variables, such as dietary lifestyle or family history of diabetes, could potentially influence our results despite accounting for known covariates. In future research endeavours, we will consider designing our own study to incorporate dietary lifestyle or family history of diabetes, and then further explore the relationship between LAP and diabetes. Lastly, our study only measured baseline WC and TG levels without considering changes over time. Future research should include more comprehensive monitoring of confounding factors, including variations in WC and TG during follow-up, to better understand the impact of LAP changes on future diabetes risk.

## Conclusion

This research revealed a non-linear positive association between LAP and DM risk, even after adjusting for multiple confounding variables. These results underscore the value of LAP as a diagnostic metric for evaluating diabetes risk, providing an economical and simple method for early detection and preventive measures.

## Electronic supplementary material

Below is the link to the electronic supplementary material.

Supplementary Material 1

Supplementary Material 2

Supplementary Material 3

Supplementary Material 4

## Data Availability

The raw data can be downloaded from the ‘DATADRYAD’ database (www.Datadryad.org). Dryad Digital Repository. https://datadryad.org/stash/dataset/doi:10.5061%2Fdryad.8q0p192.
