# Supplementary material for: Non-linear relationship between lipid accumulation products and risk of diabetes in Japanese adults
Source: Sci Rep. 2024 Nov 7;14:27106. doi: 10.1038/s41598-024-78672-0 (PMC11544252; doi:10.1038/s41598-024-78672-0)
Supplement: Supplementary file 1 — Supplementary Material 1 [file 41598_2024_78672_MOESM1_ESM.docx]

**Abbreviations**

LAP: lipid accumulation product; DM: diabetes mellitus; BMI: body mass index; WC: waist circumference; SBP: systolic blood pressure; DBP: diastolic blood pressure; ALT: alanine aminotransferase; AST: aspartate aminotransferase; GGT: gamma-glutamyl transferase; HDL-C: high-density lipoprotein cholesterol; TC: total cholesterol; TG: triglycerides; HbA1c: hemoglobin A1c; FPG: fasting plasma glucose; IR: insulin resistance; T2DM: type 2 diabetes; DM: diabetes mellitus;  HR: hazard ratio; SD: standard deviations; CI: confidence interval.
