# Supplementary figures and images for: Non-linear relationship between lipid accumulation products and risk of diabetes in Japanese adults
Source: Sci Rep. 2024 Nov 7;14:27106. doi: 10.1038/s41598-024-78672-0 (PMC11544252; doi:10.1038/s41598-024-78672-0)

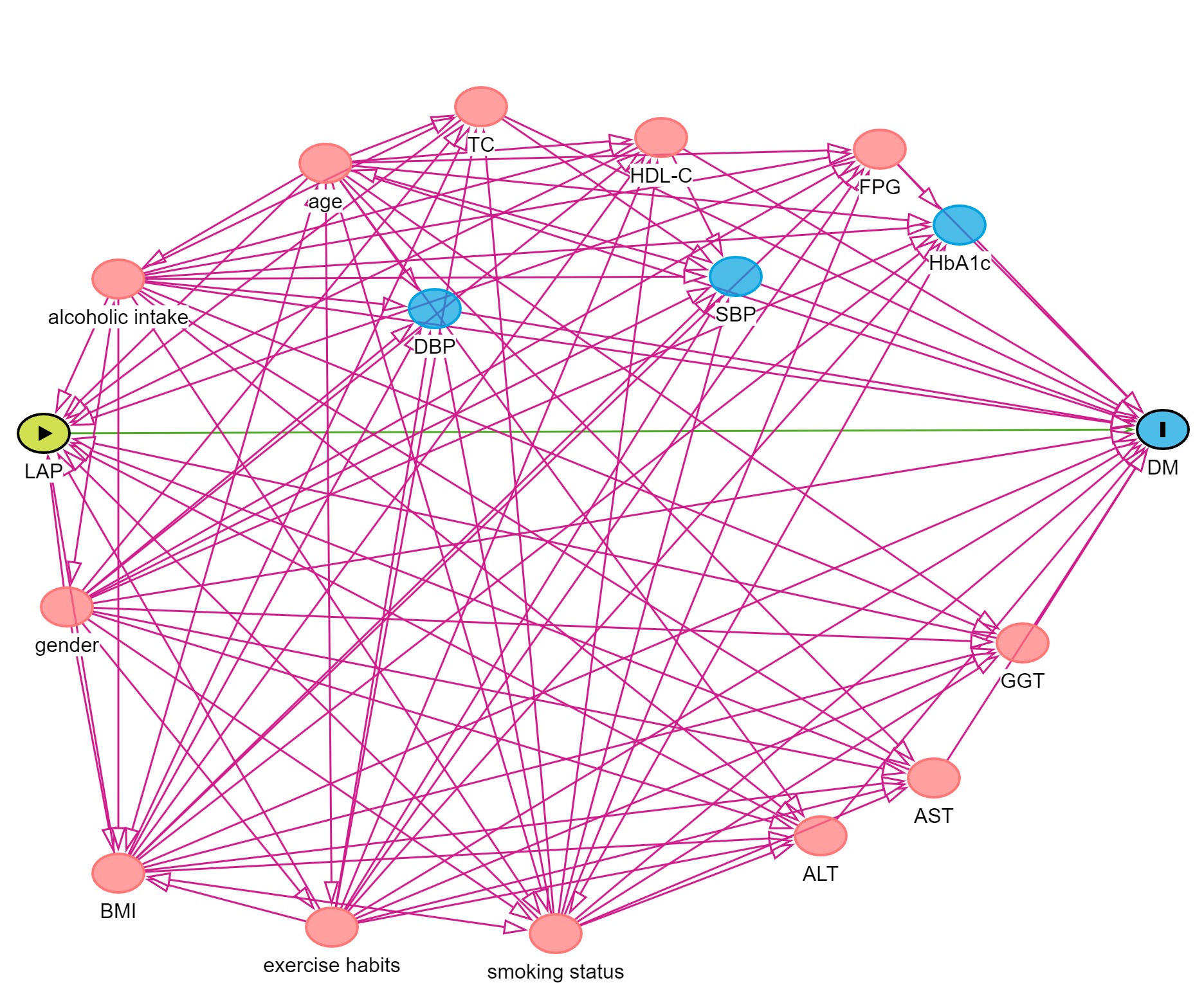

Supplement: Supplementary file 2 — Supplementary Material 2 [file 41598_2024_78672_MOESM2_ESM.jpeg]

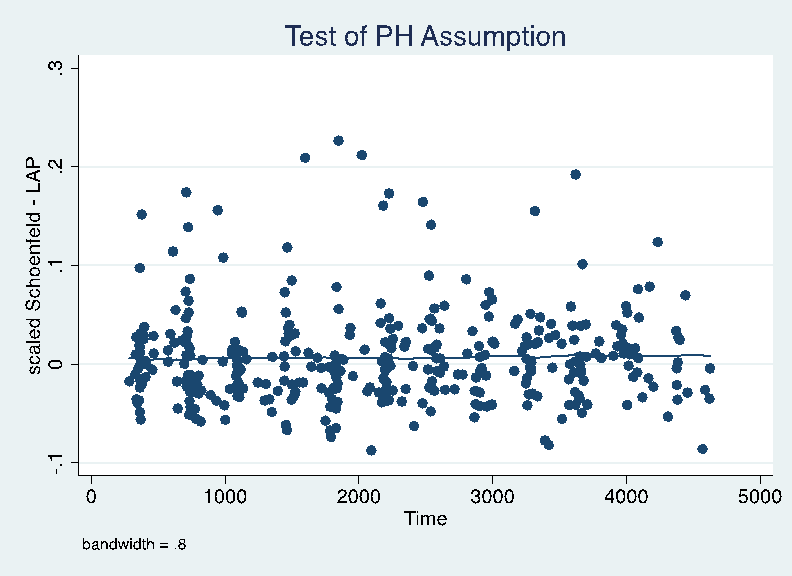

Supplement: Supplementary file 3 — Supplementary Material 3 [file 41598_2024_78672_MOESM3_ESM.png]
