# Supplementary material for: Non-linear relationship between lipid accumulation products and risk of diabetes in Japanese adults
Source: Sci Rep. 2024 Nov 7;14:27106. doi: 10.1038/s41598-024-78672-0 (PMC11544252; doi:10.1038/s41598-024-78672-0)
Supplement: Supplementary file 4 — Supplementary Material 4 [file 41598_2024_78672_MOESM4_ESM.docx]

**Table S1** The results of the collinearity screening

|  | Step 1 |
| --- | --- |
| **Gender** | 1.9 |
| **Age** | 1.2 |
| **Alcoholic intake** | 1.3 |
| **Smoking status** | 1.4 |
| **Exercise habits** | 1.0 |
| **BMI** | 1.5 |
| **ALT** | 3.9 |
| **AST** | 3.2 |
| **GGT** | 1.5 |
| **HDL-C** | 1.5 |
| **TC** | 1.3 |
| **FPG** | 1.3 |

**Table S2A** The Baseline Characteristics of male participants

| **LAP** | Q1 (LAP≤7.10) | Q2 (7.10< LAP ≤13.81) | Q3 (13.81< LAP ≤25.58) | Q4 (LAP>25.58) | P-value |
| --- | --- | --- | --- | --- | --- |
| **Participants** | 2079 | 2085 | 2088 | 2084 |  |
| **Age(years)** | 42.01 ± 9.23 | 44.31 ± 9.14 | 45.18 ± 8.80 | 44.96 ± 8.42 | <0.001 |
| **Alcoholic intake (g/wk)** | 18 (1-105) | 36 (1-126) | 22 (1-126) | 36 (1-126) | <0.001 |
| **Smoking status** |  |  |  |  | <0.001 |
| Never-smoker | 820 (39.44%) | 753 (36.12%) | 640 (30.65%) | 638 (30.61%) |  |
| Ex-smoker | 565 (27.18%) | 622 (29.83%) | 686 (32.85%) | 622 (29.85%) |  |
| Current-smoker | 694 (33.38%) | 710 (34.05%) | 762 (36.49%) | 824 (39.54%) |  |
| **Exercise habits** |  |  |  |  | <0.001 |
| No | 1589 (76.43%) | 1642 (78.75%) | 1729 (82.81%) | 1790 (85.89%) |  |
| Yes | 490 (23.57%) | 443 (21.25%) | 359 (17.19%) | 294 (14.11%) |  |
| **SBP (mmHg)** | 113.15 ± 12.48 | 117.48 ± 13.08 | 120.16 ± 13.52 | 124.86 ± 14.60 | <0.001 |
| **DBP (mmHg)** | 70.62 ± 9.03 | 73.84 ± 9.19 | 75.99 ± 9.49 | 79.36 ± 9.95 | <0.001 |
| **BMI (kg/m^2^)** | 20.48 ± 1.80 | 22.39 ± 1.85 | 23.78 ± 2.11 | 25.77 ± 2.89 | <0.001 |
| **WC (cm)** | 72.54 ± 4.13 | 78.76 ± 4.18 | 83.00 ± 4.88 | 88.37 ± 6.70 | <0.001 |
| **ALT (IU/L)** | 16 (13-21) | 19 (15-25) | 22 (17-29) | 27 (21-39) | <0.001 |
| **AST (IU/L)** | 17 (14-21) | 18 (14-21) | 19 (15-23) | 21 (17-26) | <0.001 |
| **GGT (IU/L)** | 15 (13-20) | 18 (14-25) | 21 (16-30.25) | 27 (20-41) | <0.001 |
| **HDL-C (mmol/L)** | 1.52 ± 0.37 | 1.37 ± 0.32 | 1.23 ± 0.29 | 1.09 ± 0.24 | <0.001 |
| **TG (mmol/L)** | 0.53 (0.42-0.69) | 0.76 (0.62-0.94) | 1.06 (0.88-1.31) | 1.78 (1.42-2.33) | <0.001 |
| **TC (mmol/L)** | 4.78 ± 0.77 | 5.05 ± 0.79 | 5.26 ± 0.78 | 5.56 ± 0.86 | <0.001 |
| **HbA1c (%)** | 5.11 ± 0.30 | 5.13 ± 0.30 | 5.18 ± 0.33 | 5.23 ± 0.34 | <0.001 |
| **FPG (mmol/L)** | 5.19 ± 0.37 | 5.28 ± 0.35 | 5.34 ± 0.35 | 5.43 ± 0.35 | <0.001 |
| **DM** |  |  |  |  | <0.001 |
| No | 2048 (98.51%) | 2050 (98.32%) | 2032 (97.32%) | 1920 (92.13%) |  |
| Yes | 31 (1.49%) | 35 (1.68%) | 56 (2.68%) | 164 (7.87%) |  |
| **Cumulative incidence** (%) (95% CI) | 1.49 (0.97-2.01) | 1.68 (1.13-2.23) | 2.68 (1.99-3.38) | 7.87 (6.71-9.03) | <0.001 |
| **Per 100,000 person-year** | 252.66 | 272.80 | 423.33 | 1,237.51 | <0.001 |

Values are presented as n (%) or mean ± SD or median (quartile)

LAP: lipid accumulation product; BMI: body mass index; WC: waist circumference; SBP: systolic blood pressure; DBP: diastolic blood pressure; ALT: alanine aminotransferase; AST: aspartate aminotransferase; GGT: gamma-glutamyl transferase; HDL-C: high-density lipoprotein cholesterol; TC: total cholesterol; TG: triglycerides; HbA1c: hemoglobin A1c; FPG: fasting plasma glucose; CI: confidence interval; DM: diabetes mellitus.

**Table S2B** The Baseline Characteristics of female participants

| **LAP** | Q1 (LAP≤3.60) | Q2 (3.60< LAP ≤6.75) | Q3 (6.75< LAP ≤12.70) | Q4 (LAP>12.70) | P-value |
| --- | --- | --- | --- | --- | --- |
| **Participants** | 1731 | 1731 | 1731 | 1734 |  |
| **Age(years)** | 39.87 ± 8.07 | 41.61 ± 8.14 | 44.26 ± 8.28 | 47.33 ± 8.56 | <0.001 |
| **Alcoholic intake (g/wk)** | 1 (0-2.8) | 1 (0-12) | 1 (0-12) | 1 (0-2.8) | 0.006 |
| **Smoking status** |  |  |  |  | 0.016 |
| Never-smoker | 1532 (88.50%) | 1525 (88.10%) | 1493 (86.25%) | 1497 (86.33%) |  |
| Ex-smoker | 96 (5.55%) | 119 (6.87%) | 119 (6.87%) | 103 (5.94%) |  |
| Current-smoker | 103 (5.95%) | 87 (5.03%) | 119 (6.87%) | 134 (7.73%) |  |
| **Exercise habits** |  |  |  |  | 0.121 |
| No | 1432 (82.73%) | 1480 (85.50%) | 1456 (84.11%) | 1474 (85.01%) |  |
| Yes | 299 (17.27%) | 251 (14.50%) | 275 (15.89%) | 260 (14.99%) |  |
| **SBP (mmHg)** | 103.78 ± 11.92 | 106.43 ± 12.21 | 109.83 ± 13.30 | 117.84 ± 15.39 | <0.001 |
| **DBP (mmHg)** | 64.27 ± 8.16 | 65.67 ± 8.57 | 67.81 ± 9.35 | 73.07 ± 10.41 | <0.001 |
| **BMI (kg/m^2^)** | 18.84 ± 1.57 | 20.11 ± 1.70 | 21.38 ± 2.00 | 23.93 ± 3.23 | <0.001 |
| **WC (cm)** | 64.25 ± 3.71 | 69.19 ± 3.92 | 73.55 ± 4.82 | 80.66 ± 7.22 | <0.001 |
| **ALT (IU/L)** | 13 (10-16) | 13 (11-16) | 13 (10-17) | 16 (12-20) | <0.001 |
| **AST (IU/L)** | 16 (13-19) | 16 (13-19) | 16 (13-19) | 17 (14-20) | <0.001 |
| **GGT (IU/L)** | 11 (9-13) | 11 (9-14) | 12 (10-14) | 13 (11-18) | <0.001 |
| **HDL-C (mmol/L)** | 1.78 ± 0.37 | 1.72 ± 0.36 | 1.64 ± 0.37 | 1.44 ± 0.34 | <0.001 |
| **TG (mmol/L)** | 0.37 (0.27-0.49) | 0.46 (0.38-0.60) | 0.61 (0.50-0.76) | 0.98 (0.79-1.28) | <0.001 |
| **TC (mmol/L)** | 4.74 ± 0.78 | 4.93 ± 0.79 | 5.14 ± 0.85 | 5.53 ± 0.88 | <0.001 |
| **HbA1c (%)** | 5.10 ± 0.30 | 5.13 ± 0.30 | 5.19 ± 0.31 | 5.32 ± 0.33 | <0.001 |
| **FPG (mmol/L)** | 4.86 ± 0.36 | 4.91 ± 0.37 | 5.00 ± 0.38 | 5.18 ± 0.39 | <0.001 |
| **DM** |  |  |  |  | <0.001 |
| No | 1725 (99.65%) | 1726 (99.71%) | 1719 (99.31%) | 1670 (96.31%) |  |
| Yes | 6 (0.35%) | 5 (0.29%) | 12 (0.69%) | 64 (3.69%) |  |
| **Cumulative incidence** (%) (95% CI) | 0.35 (0.06-0.62) | 0.29 (0.04-0.54) | 0.69 (0.30-1.08) | 3.69 (2.80-4.58) | <0.001 |
| **Per 100,000 person-year** | 55.19 | 48.16 | 121.75 | 667.86 | <0.001 |

Values are presented as n (%) or mean ± SD or median (quartile)

LAP: lipid accumulation product; BMI: body mass index; WC: waist circumference; SBP: systolic blood pressure; DBP: diastolic blood pressure; ALT: alanine aminotransferase; AST: aspartate aminotransferase; GGT: gamma-glutamyl transferase; HDL-C: high-density lipoprotein cholesterol; TC: total cholesterol; TG: triglycerides; HbA1c: hemoglobin A1c; FPG: fasting plasma glucose.

**Table S3** The results of the univariate analysis results of risk factors associated with diabetes

|  | **N (%)/M±SD** | **HR (95% CI)** | ***P* value** |
| --- | --- | --- | --- |
| **Sex** |  |  | <0.0001 |
| Female | 6927 (45.38%) | Ref. |  |
| Male | 8336 (54.62%) | 2.50 (1.97, 3.18) |  |
| **Age(years)** | 43.73 ± 8.88 | 1.06 (1.04, 1.07) | <0.0001 |
| **Alcoholic intake (g/wk)** | 48.00 ± 82.53 | 1.00 (1.00, 1.00) | 0.0016 |
| **Smoking status** |  |  |  |
| Never-smoker | 8898 (58.30%) | Ref. |  |
| Ex-smoker | 2932 (19.21%) | 1.63 (1.24, 2.16) | 0.0005 |
| Current-smoker | 3433 (22.49%) | 2.58 (2.05, 3.24) | <0.0001 |
| **Exercise habits** |  |  | 0.0656 |
| No | 12592 (82.50%) | Ref. |  |
| Yes | 2671 (17.50%) | 0.76 (0.56, 1.02) |  |
| **SBP (mmHg)** | 114.63 ± 14.94 | 1.03 (1.03, 1.04) | <0.0001 |
| **DBP (mmHg)** | 71.67 ± 10.50 | 1.05 (1.04, 1.06) | <0.0001 |
| **BMI (kg/m^2^)** | 22.18 ± 3.10 | 1.24 (1.21, 1.27) | <0.0001 |
| **WC (cm)** | 76.70 ± 8.93 | 1.09 (1.08, 1.10) | <0.0001 |
| **ALT (IU/L)** | 76.70 ± 8.93 | 1.09 (1.08, 1.10) | <0.0001 |
| **AST (IU/L)** | 20.03 ± 14.37 | 1.01 (1.01, 1.01) | <0.0001 |
| **GGT (IU/L)** | 18.40 ± 8.63 | 1.01 (1.01, 1.01) | <0.0001 |
| **HDL-C (mmol/L)** | 1.46 ± 0.40 | 0.15 (0.11, 0.21) | <0.0001 |
| **TG (mmol/L)** | 0.92 ± 0.66 | 1.79 (1.67, 1.92) | <0.0001 |
| **TC (mmol/L)** | 5.13 ± 0.86 | 1.49 (1.34, 1.66) | <0.0001 |
| **HbA1c (%)** | 5.17 ± 0.32 | 55.55 (40.33, 76.52) | <0.0001 |
| **FPG (mmol/L)** | 5.16 ± 0.41 | 25.17 (18.55, 34.16) | <0.0001 |
| **LAP** | 15.33 ± 16.64 | 1.03 (1.03, 1.03) | <0.0001 |

N (%)/M±SD: Values are presented n (%) or mean ± SD

LAP: lipid accumulation product; BMI: body mass index; WC: waist circumference; SBP: systolic blood pressure; DBP: diastolic blood pressure; ALT: alanine aminotransferase; AST: aspartate aminotransferase; GGT: gamma-glutamyl transferase; HDL-C: high-density lipoprotein cholesterol; TC: total cholesterol; TG: triglycerides; HbA1c: hemoglobin A1c; FPG: fasting plasma glucose; HR: hazard ratio; CI: confidence interval; Ref: Reference.

**Table S4** Results of Proportional Hazards Assumption Testing

| **Variable** | **rho** | **chi-square** | **P value** |
| --- | --- | --- | --- |
| **LAP** | 0.014 | 0.070 | 0.788 |
| **Gender** | -0.093 | 3.770 | 0.052 |
| **Age** | -0.022 | 0.200 | 0.656 |
| **Alcoholic intake** | -0.062 | 1.570 | 0.210 |
| **Smoking status** | 0.057 | 1.410 | 0.235 |
| **Exercise habits** | -0.092 | 3.110 | 0.078 |
| **BMI** | -0.037 | 0.570 | 0.451 |
| **ALT** | 0.052 | 1.080 | 0.299 |
| **AST** | -0.059 | 1.270 | 0.259 |
| **GGT** | 0.072 | 1.950 | 0.163 |
| **HDL-C** | 0.074 | 2.150 | 0.143 |
| **TC** | -0.002 | 0.000 | 0.968 |
| **FPG** | -0.057 | 1.570 | 0.210 |
| **Global Test** |  | 20.600 | 0.081 |

**Table S5** Relationship between LAP and incident diabetes in different sensitivity analyses

| Variable | Model 5 (HR,95%CI, P) | Model 6 (HR,95% CI, P) |
| --- | --- | --- |
| **Total**  LAP | 1.01 (1.00, 1.02) 0.0006 | 1.01 (1.00, 1.02) 0.0111 |
| **Total**  LAP (quartile) |  |  |
| Q1 | Ref. | Ref. |
| Q2 | 0.67 (0.39, 1.13) 0.1346 | 0.90 (0.36, 2.23) 0.8211 |
| Q3 | 0.59 (0.35, 0.98) 0.0436 | 1.12 (0.49, 2.57) 0.7897 |
| Q4 | 0.68 (0.38, 1.20) 0.1801 | 1.25 (0.51, 3.06) 0.6245 |
| P for trend | 0.4398 | 0.4313 |
| **Female**  LAP | 1.02 (1.01, 1.03) 0.0033 | 1.00 (0.99, 1.02) 0.6692 |
| **Female**  LAP (quartile) |  |  |
| Q1 |  | Ref. |
| Q2 | 0.64 (0.19, 2.13) 0.4690 | 1.90 (0.34, 10.50) 0.4641 |
| Q3 | 0.93 (0.34, 2.59) 0.8929 | 1.38 (0.26, 7.47) 0.7080 |
| Q4 | 1.65 (0.59, 4.63) 0.3445 | 2.72 (0.49, 15.03) 0.2501 |
| P for trend |  | 0.2749 |
| **Male**  LAP | 1.01 (1.00, 1.01) 0.0104 | 1.01 (1.00, 1.02) 0.0228 |
| **Male**  LAP (quartile) |  |  |
| Q1 | Ref. | Ref. |
| Q2 | 0.55 (0.32, 0.93) 0.0253 | 1.52 (0.54, 4.29) 0.4291 |
| Q3 | 0.50 (0.29, 0.84) 0.0097 | 1.47 (0.53, 4.06) 0.4598 |
| Q4 | 0.72 (0.40, 1.27) 0.2538 | 1.86 (0.65, 5.30) 0.2457 |
| P for trend | 0.8588 | 0.2524 |

Model 5 was sensitivity analysis after excluding those with elevated blood pressure (SBP ≥140 mmHg or DBP ≥90 mmHg). We adjusted sex, age, alcoholic intake, smoking status, exercise habits, BMI, ALT, AST, GGT, HDL-C, TC, and FPG.

Model 6 was sensitivity analysis after excluding those with age≥45 years. We adjusted sex, age, alcoholic intake, smoking status, exercise habits, BMI, ALT, AST, GGT, HDL-C, TC, and FPG.

Note: The models were not adjusted for sex variables in both male and female models.

HR: hazard ratio; CI: confidence interval; Ref.: Reference; LAP: lipid accumulation product.

**Table S6** Effect size of LAP on diabetes in prespecified and exploratory subgroups

| Characteristic | No of patients | Effect size(95%CI) | P value | P for interaction |
| --- | --- | --- | --- | --- |
| **Sex** |  |  |  | 0.1086 |
| Female | 6927 | 1.01 (1.00, 1.02) | 0.0035 |  |
| Male | 8336 | 1.01 (1.00, 1.01) | 0.0264 |  |
| **Alcoholic intake (g/wk)** |  |  |  | 0.0128 |
| =0 | 4651 | 1.02 (1.01, 1.03) | 0.0002 |  |
| >0 | 10612 | 1.00 (1.00, 1.01) | 0.3650 |  |
| **Smoking status** |  |  |  | 0.6210 |
| Never-smoker | 8898 | 1.01 (1.00, 1.02) | 0.0377 |  |
| Ex-smoker | 2932 | 1.01 (1.00, 1.02) | 0.0729 |  |
| Current-smoker | 3433 | 1.00 (1.00, 1.01) | 0.2936 |  |
| **Exercise habits** |  |  |  | 0.6671 |
| No | 12592 | 1.01 (1.00, 1.01) | 0.0165 |  |
| Yes | 2671 | 1.01 (1.00, 1.02) | 0.1740 |  |
| **SBP (mmHg)** |  |  |  | 0.0913 |
| <140 | 14480 | 1.01 (1.00, 1.02) | 0.0007 |  |
| ≥140 | 783 | 1.00 (0.99, 1.01) | 0.7446 |  |
| **DBP (mmHg)** |  |  |  | 0.0300 |
| <90 | 14501 | 1.01 (1.00, 1.02) | 0.0006 |  |
| ≥90 | 762 | 0.99 (0.98, 1.01) | 0.3404 |  |

Note 1: The above model adjusted for we adjusted forsex, age, alcoholic intake, smoking status, exercise habits, BMI, ALT, AST, GGT, HDL-C, TC, and FPG.

Note 2: The model is not adjusted for the stratification variable in each case.

**Table S7** Relationship between LAP and incident diabetes in participants of different ages.

| Variable | Model 7 (HR,95%CI, P) | Model 8 (HR,95% CI, P) | Model 9 (HR,95% CI, P) | Model 10 (HR,95% CI, P) |
| --- | --- | --- | --- | --- |
| **Total**  LAP | 1.03 (1.02, 1.03) <0.0001 | 1.03 (1.02, 1.03) <0.0001 | 1.02 (1.01, 1.02) <0.0001 | 1.01 (1.00, 1.01) 0.0042 |
| **Total**  LAP (quartile) |  |  |  |  |
| Q1 | Ref. | Ref. | Ref. | Ref. |
| Q2 | 1.35 (0.80, 2.29) 0.2631 | 1.24 (0.73, 2.11) 0.4202 | 0.86 (0.50, 1.46) 0.5760 | 0.67 (0.39, 1.14) 0.1416 |
| Q3 | 2.53 (1.57, 4.07) 0.0001 | 2.17 (1.34, 3.49) 0.0015 | 1.10 (0.67, 1.81) 0.7019 | 0.65 (0.39, 1.09) 0.1008 |
| Q4 | 7.87 (5.07, 12.21) <0.0001 | 6.46 (4.15, 10.04) <0.0001 | 2.02 (1.23, 3.33) 0.0059 | 0.78 (0.45, 1.36) 0.3858 |
| P for trend | <0.0001 | <0.0001 | <0.0001 | 0.9108 |
| **Young individuals**  LAP | 1.03 (1.03, 1.04) <0.0001 | 1.03 (1.03, 1.03) <0.0001 | 1.02 (1.01, 1.02) <0.0001 | 1.01 (1.00, 1.02) 0.0111 |
| LAP (quartile) |  |  |  |  |
| Q1 | Ref. | Ref. | Ref. | Ref. |
| Q2 | 1.82 (0.74, 4.44) 0.1908 | 1.75 (0.72, 4.29) 0.2190 | 1.21 (0.49, 2.97) 0.6801 | 0.90 (0.36, 2.23) 0.8211 |
| Q3 | 5.18 (2.38, 11.27) <0.0001 | 4.68 (2.14, 10.25) 0.0001 | 2.23 (1.00, 4.97) 0.0501 | 1.12 (0.49, 2.57) 0.7897 |
| Q4 | 17.65 (8.60, 36.25) <0.0001 | 15.87 (7.64, 32.98) <0.0001 | 4.15 (1.85, 9.28) 0.0005 | 1.25 (0.51, 3.06) 0.6245 |
| P for trend | <0.0001 | <0.0001 | <0.0001 | 0.4313 |
| **Middle-aged individuals**  LAP | 1.03 (1.02, 1.03) <0.0001 | 1.02 (1.02, 1.03) <0.0001 | 1.01 (1.01, 1.02) <0.0001 | 1.01 (1.00, 1.01) 0.0459 |
| LAP (quartile) |  |  |  |  |
| Q1 | Ref. | Ref. | Ref. | Ref. |
| Q2 | 1.00 (0.52, 1.91) 0.9895 | 0.92 (0.48, 1.77) 0.8079 | 0.67 (0.34, 1.29) 0.2303 | 0.54 (0.27, 1.06) 0.0743 |
| Q3 | 1.37 (0.76, 2.47) 0.2997 | 1.23 (0.68, 2.23) 0.5006 | 0.69 (0.37, 1.30) 0.2548 | 0.49 (0.25, 0.95) 0.0355 |
| Q4 | 4.21 (2.50, 7.09) <0.0001 | 3.47 (2.04, 5.90) <0.0001 | 1.33 (0.71, 2.51) 0.3776 | 0.66 (0.32, 1.36) 0.2589 |
| P for trend | <0.0001 | <0.0001 | 0.0284 | 0.97 (0.77, 1.22) 0.7932 |

Model 7: we did not adjust for any covariants.

Model 8: we adjusted for sex, age, alcoholic intake, smoking status, and exercise habits.

Model 9: we adjusted for sex, age, alcoholic intake, smoking status, exercise habits, and BMI.

Model 10: we adjusted for sex, age, alcoholic intake, smoking status, exercise habits, BMI, ALT, AST, GGT, HDL-C, TC, and FPG.

HR: hazard ratio; CI: confidence interval; Ref.: Reference; LAP: lipid accumulation product.

**Table S8** Relationship between LAP and incident diabetes in different models

| Variable | Model 11 (HR,95% CI, P) |
| --- | --- |
| **Total**  LAP | 1.01 (1.00, 1.01) 0.0014 |
| **Total**  LAP (quartile) |  |
| Q1 | Ref. |
| Q2 | 0.65 (0.39, 1.09) 0.0991 |
| Q3 | 0.67 (0.41, 1.09) 0.1094 |
| Q4 | 0.71 (0.41, 1.20) 0.2013 |
| P for trend | 0.5542 |
| **Female**  LAP | 1.01 (1.00, 1.01) 0.0099 |
| **Female**  LAP (quartile) |  |
| Q1 | Ref. |
| Q2 | 0.58 (0.35, 0.95) 0.0323 |
| Q3 | 0.50 (0.30, 0.82) 0.0065 |
| Q4 | 0.72 (0.42, 1.21) 0.2145 |
| P for trend | 0.8289 |
| **Male**  LAP | 1.02 (1.00, 1.03) 0.0076 |
| **Male**  LAP (quartile) |  |
| Q1 | Ref. |
| Q2 | 0.74 (0.22, 2.47) 0.6290 |
| Q3 | 1.07 (0.39, 2.97) 0.8978 |
| Q4 | 1.88 (0.68, 5.25) 0.2261 |
| P for trend | 0.0881 |

Model 11: we adjusted for sex, age, alcoholic intake, smoking status, exercise habits, SBP, DBP, BMI, ALT, AST, GGT, HDL-C, TC, HbA1c, and FPG.

Note: The models were not adjusted for sex variables in both male and female models.

HR: hazard ratio; CI: confidence interval; Ref.: Reference; LAP: lipid accumulation product.
